# MicroRNAs in Kawasaki disease: An update on diagnosis, therapy and monitoring

**DOI:** 10.3389/fimmu.2022.1016575

**Published:** 2022-10-24

**Authors:** Yiyi Xiong, Jiawei Xu, Deju Zhang, Shuqin Wu, Zhangwang Li, Jing Zhang, Zhongbin Xia, Panpan Xia, Cai Xia, Xiaoyi Tang, Xiao Liu, Jianping Liu, Peng Yu

**Affiliations:** ^1^ The Second Clinical Medical College of Nanchang University, The Second Affiliated Hospital of Nanchang University, Nanchang, China; ^2^ Food and Nutritional Sciences, School of Biological Sciences, The University of Hong Kong, Hong Kong, Hong Kong SAR, China; ^3^ Department of Anesthesiology, The Second Affiliated Hospital of Nanchang University, Nanchang, China; ^4^ Department of Endocrinology and Metabolism, The Second Affiliated Hospital of Nanchang University, Nanchang, Jiangxi, China; ^5^ Department of Cardiology, The Second Affiliated Hospital of Sun Yat-Sen University, Guangzhou, China

**Keywords:** Kawasaki disease, micriRNAs, biomarker, therapeutic target, prognosis

## Abstract

Kawasaki disease (KD) is an acute autoimmune vascular disease featured with a long stage of febrile. It predominantly afflicts children under 5 years old and causes an increased risk of cardiovascular combinations. The onset and progression of KD are impacted by many aspects, including genetic susceptibility, infection, and immunity. In recent years, many studies revealed that miRNAs, a novel class of small non-coding RNAs, may play an indispensable role in the development of KD *via* differential expression and participation in the central pathogenesis of KD comprise of the modulation of immunity, inflammatory response and vascular dysregulation. Although specific diagnose criteria remains unclear up to date, accumulating clinical evidence indicated that miRNAs, as small molecules, could serve as potential diagnostic biomarkers and exhibit extraordinary specificity and sensitivity. Besides, miRNAs have gained attention in affecting therapies for Kawasaki disease and providing new insights into personalized treatment. Through consanguineous coordination with classical therapies, miRNAs could overcome the inevitable drug-resistance and poor prognosis problem in a novel point of view. In this review, we systematically reviewed the existing literature and summarized those findings to analyze the latest mechanism to explore the role of miRNAs in the treatment of KD from basic and clinical aspects retrospectively. Our discussion helps to better understand the pathogenesis of KD and may offer profound inspiration on KD diagnosis, treatment, and prognosis.

## 1 Introduction

Kawasaki disease, which was firstly defined as a mucocutaneous lymph node syndrome, is an acute and autoimmune disease with febrile vasculitis (1, 2). The main patient population of KD includes children under 5 years old and infants about 6-18 months. Generally, KD has one imperative feature: pyrexia over five days and four concomitant symptoms: extreme changes such as desquamation and erythematous rash, bilateral conjunctival injection, oral changes including cracked lips or strawberry tongue and cervical lymphadenopathy over 1.5cm in diameter (3–5). Although KD used to be considered as a self-limited vasculitis prominently impacting medium-sized and small-sized vessels, nowadays plenty of studies have elucidated that severe Kawasaki disease without treatment or with a course of treatment over 10 days has a tight association with increased occurrence of coronary artery aneurisms (CAA) (6). Kawasaki disease remains the primary reason for acquired heart disease in children in developed countries, bringing unbearable tragedies to tens of thousands of families.

For a long time, a large number of researches have focused on the pathogenesis of Kawasaki disease. The mainstream theories are from 3 perspectives: genetic susceptibility, infection and immune system. Distinct genetic background has already revealed essential role in affecting the risk of Kawasaki disease onset. The epidemiological analysis of KD indicated that the prevalence rate is extremely higher in Asia, about 2.5 times more than in the west (7). KD has a typical ethnic distribution feature, especially in Japanese and children of Japanese ancestry (8, 9). Emerging genome-wide association studies (GWAS) from the immunogenetics has categorized representative susceptible genes such as B cell lymphoid kinase (BLK), Caspase 3(CASP3), Fc fragment of IgG receptor IIa (FCGR2A) and so on into 4 major fields including of decreased apoptosis, enhanced T cell activity, dysregulated B cell signaling and altered TGF-β signaling (10, 11). Effective evidences standing by immunity are abundant. The central pathogenesis of KD involves of dysregulation of both innate immune and adaptive immune, which subsequently elevate the risk of cardiovascular artery abnormities (CAA) (10). Besides, recent studies regarding seasonal fluctuation, low recurrence risk and epidemiological features, on one hand, indicate the influence of infection on Kawasaki disease, on the other hand, reveal the presence of multiple possible pathogens that are highly correlated with KD. For example, plenty of evidences have indicated that the Sars-Cov-2 virus might be a “priming trigger” that leads to KD (12). Although numerous studies have been conducted to investigate into the pathogenesis, no specific mechanism has been elucidated until today. Therefore, exploring the mechanism from a novel perspective is of significance.

MicroRNAs, a class of small non-coding RNA known for their function of regulating genes indirectly in post-transcriptional level targeting in specific mRNA. In 2013, Shimizu C et al. firstly reported that miRNA led to Kawasaki disease (13). Subsequent studies revealed that miRNAs could play a key role in the pathophysiology of Kawasaki disease (14). MiRNAs mainly affect the advance of Kawasaki disease by affecting the susceptibility of KD, regulating inflammation and immunity progress, and causing vascular endothelial dysfunction (15, 16). Both basic and clinical studies are emerging rapidly supporting that the differentially expressed miRNAs were involved in the pathogenesis of KD and served as its predictors (17, 18). Except for the exploration of KD mechanism, many studies also revealed the potential of miRNAs as prognostic biomarkers and therapeutic targets of Kawasaki disease. Until now, the gold standard therapy for KD is intravenous immunoglobulin (IVIG) with Aspirin, where no significant steps ahead have been moved since 1980s. Clinical outcome presented that about 80% of patients could recover without much sequelae, however, the remanent patients showed resistance to this classical therapy and had more complications, poorer prognosis and higher death rates. It’s also a worrying phenomenon that 25.8% of patients reported adverse reactions which haven’t been ameliorated until today (19). Interestingly, miRNA seems to play a role in improving the adverse reaction and reducing resistance. Emerging evidence brought forward that miRNAs show apparent variation during the treatment course (20). Besides, miRNAs showed extraordinary effects in identifying drug resistant patients to help clinicians quickly formulate personalized treatment arrangements (21). Therefore, in this review, we summarized the newest role of miRNA in monitoring, diagnosing and treating KD in order to inspire new ideas for KD.

## 2 Current concepts in the etiology and pathogenesis of Kawasaki disease

### 2.1 Genetics

**Figure 1:**
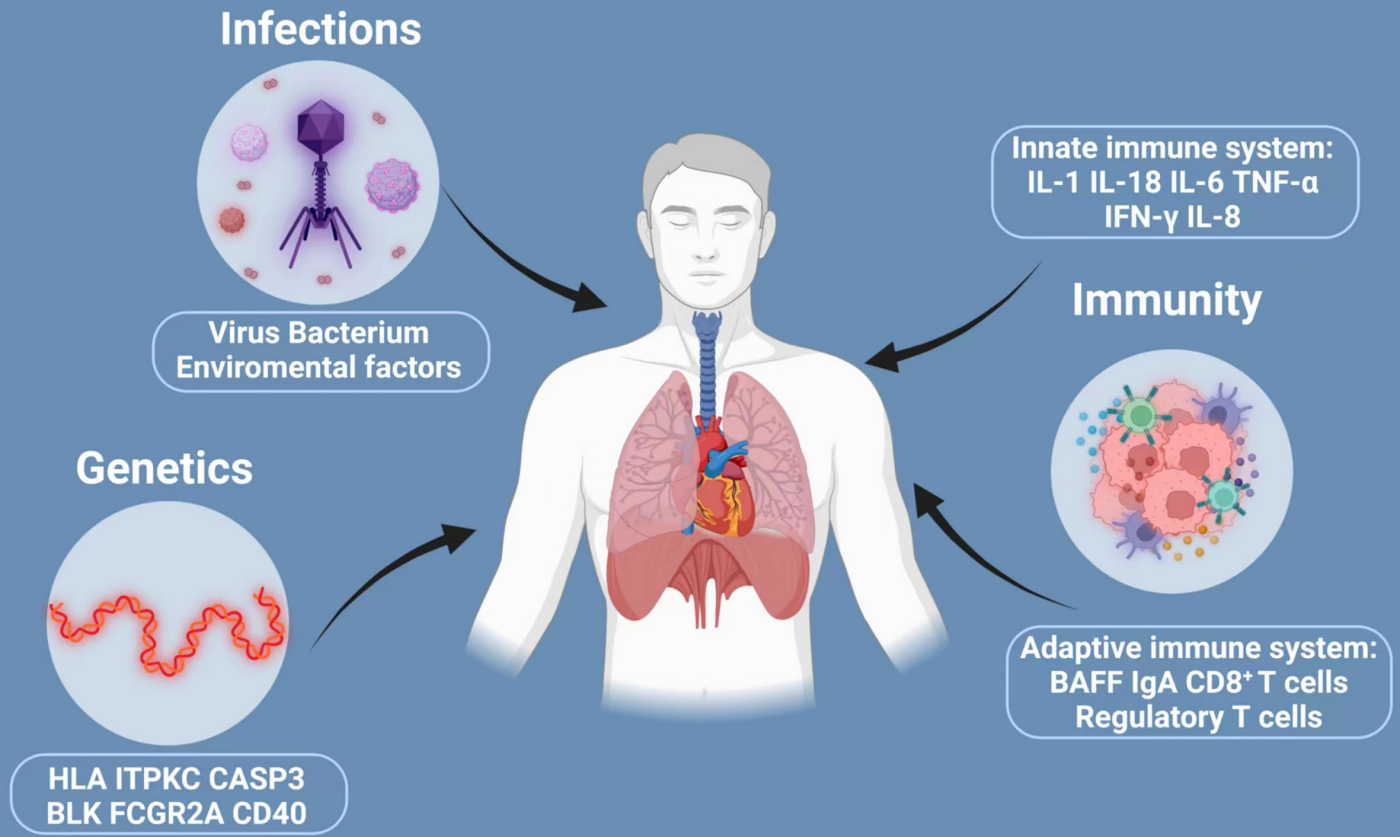
Three major factors implicated in the development of Kawasaki disease: infections, genetics and immunity. The development and progression of KD is a concerted effect of multifaceted contributions, mainly including genetic susceptibility, infectious trigger and immunological factors. However, many of the claims are unproved. The current understanding is that KD is triggered in a small population of genetically susceptible children by a ubiquitous infection that trigger inflammatory cascades with activation of both the innate and adaptive immune system and finally may cause KD. HLA, human leukocyte antigen; ITPKC, inositol 1,4,5-Trisphosphate kinase-C; CASP3, caspase3; BLK, B cell lymphoid kinase; FCGR2A, Fc fragment of IgG receptor IIa; IL-1, interleukin-2; IL-18, interleukin-18; IL-6, interleukin-6; TNF-α, transforming growth factor-α; IFN-γ, interferon-γ; IL-8, interleukin-8; BAFF, B-cell Activating factor of the TNF family.

All along, the etiology and pathogenesis of KD have been studied extensively, yet not fully understood. Past research has shown that the development and progression of KD is a concerted effect of multifaceted contributions, mainly including genetic susceptibility, infectious trigger and immunological factors. We have shown how these factors interact and contribute to the pathogenesis of KD in **Figure 1**. Family linkage studies, genome-wide association studies and subsequent validation studies implicated that several genes are consistently associated with KD, including Human Leukocyte Antigen (HLA), inositol 1,4,5-Trisphosphate kinase-C ITPKC, CASP3, BLK, Fc fragment of IgG receptor IIa (FCGR2A) and CD40 (22–24), which provide new directions for future research on KD pathogenesis.

Initial research on KD-related genes was centered on HLA, which is linked with the susceptibility to many autoimmune diseases (25). Results found that HLA-DRB1, -B5, -Bw51 and -Bw44 were closely associated with KD susceptibility (25). There are various associations of HLA among different ethnic groups. A genome-wide association study by Onouchi demonstrated an HLA determinant that affected susceptibility in Japanese and Taiwanese patients but not in patients with European descent (26). A previous study performed in Japan found that HLA-Bw22 was more frequently detected in KD patients, and another study in the United States showed that HLA-Bw22 was highly associated with HLA-Bw51, another potential protective allele (27, 28). HLA class I and II may have contributed to antigen presentation and act as activation markers for immune cells (26). In a human study from KD patients by Shimizu et al., they showed that CD8+ T cells found in the intima of coronary artery aneurysms stain positive for HLA-DR, which has a relation to inflammation and activated phenotype (29). The ITPKC gene codes for one of the three isoenzymes of inositol 1,4,5-triphosphate 3 kinase, which is a second messenger implied in the Ca++/NFAT signal pathway (22). ITPKC is a known negative regulator of T cell activation and its polymorphism results in enhanced activation of T cells and increased expression of cytokines, such as interleukin-2(IL-2) (30). That can lead to more prolonged expansion of pro-inflammatory T cells during the acute phase, which may cause vascular endothelial damage and contribute to KD development and augment of disease severity (31, 32). It is worth mentioning that the incidence of ITPKC genetic polymorphisms is significantly different, which needs further investigation (33). Caspase3 (CASP3) has been shown to participate in the Activation Induced Cell Death (AICD) apoptosis pathway, which regulates the apoptosis of immature cells (31). Meanwhile, it is also the positive regulator of the Ca2+/NFAT pathway to cleave type 1 Inositol 1,4,5 triphosphate receptor in apoptotic T cells (34). Researches have suggested that the G to A substitution within exon 1 of CASP3 reduces the binding of NFAT with DNA around the SNP, and decreases the mRNA transcription of CASP3 (33). It inhibits T-cell apoptosis, causes the long-term activation of immune cells, and increases cytokine expression, which finally increases the susceptibility of KD (22). In addition, it is worth mentioning that no significant correlation was found between the CASP3 polymorphism and the risk of CAAs or response to IVIG treatment (31). In Japanese, Taiwanese, Korean and Asian populations in the USA, several studies revealed that BLK SNPs have also been implicated in KD (25). BLK is a tyrosine kinase which mainly expresses in B cells (25). There has considerable relevance between KD and FAM167A-BLK region on chromosome 8p23-p22, which was confirmed by two independent GWAS studies in Japan and Taiwan. Furthermore, BLK was found to be necessary for the maturation of IL-17 producing γδT cells in animal studies (25). And IL-17 expression levels were elevated in the acute phase of KD due to BLK polymorphism (25). These findings point to one important conclusion: B cell is promisingly involved in KD pathogenesis. Another gene found to be closely tied to KD is FCGR2A, the Fc fragment of IgG (33). FCGR2A is expressed on the surface of immune cells, like dendritic cells, macrophages, monocytes and neutrophils and transduces the activation signals into cells on binding to immune complexes (ICs) (35). One research proposed that the signal connection between FCGR2A and ICs might cause immune activation during KD acute phase (36). It is identified to have two susceptibility loci, including one polymorphism in the FCGR2A gene (37). Data suggested that the polymorphisms in FCGR2A may have different effects on different ethnicities (38). Besides, it should be noted that the high level of FcγRIIA mRNA expression is associated with IVIG resistance (39). However, the mechanism by which IVIG works on STING is not clear. This was also found in two GWAS studies by Japanese (23) and Taiwanese (40) that KD is associated with the SNPs around CD40 located on chromosome 20q12-q13.2. CD40 is expressed on B cells, macrophages, endothelial cells, epithelial cells, smooth muscle cells, fibroblasts, and adipocytes and its receptor (41). Research indicates that CD40L expression rises rapidly during the KD acute phase. The upregulation of CD40-CD40L signaling might promote KD progression, which would hopefully become a potential new molecular target for KD therapy (25, 42).

As discussed above, we can draw the conclusion that KD susceptibility and the disease outcome were influenced by genetic factors and interrelated signaling pathways, including aneurysm formation and response to IVIG. And different populations may have diverse genetic polymorphism.

### 2.2 Infection

One of the epidemiologic features of KD is infection, especially asymptomatic infection. And it may cause KD in a small population of genetically susceptible children.

There are several observations supporting this hypothesis. First, one apparent reason is the seasonality of KD. The number of patients reaches a consistent peak reported in January and gradually increases from March to June roughly (43). Seasonal fluctuation in connection to infectious pathogens has been reported in previous studies, especially viral pathogens (41). Another supporting evidence is related to tropospheric wind patterns whose presence in different locations may coincide with the incidence of KD, which may carry environmental toxins or an infectious pathogen (44, 45). In addition, the clinical features of KD overlap with those of other infectious agents greatly. The most obvious clinical features are scarlet fever, multisystem inflammatory syndrome and adenovirus (46). What’s more, there is a monomorphic distribution in the onset of KD with the highest incidence rate occurring in babies aged 9 to 11 months, then gradually reducing with the increase in age (47). This indicates that there may exist protective transplacental antibodies against infection, which would be weakened gradually in the first few months after babies are born (48). Finally, some cases suggested that the risk of KD in a child would increase sketchy tenfold if a sibling gets KD. Nagao et al. also reported that KD may have a similar pattern of transmission to infections after contact with other infected individuals (49).

Two causes are currently considered: viral etiology and bacterial superantigens. Viral etiology is based on the following points. KD patients failed to respond to antibiotic therapy. Furthermore, it can be speculated that virus is a major cause of KD, because of the extensive infiltration of CD8+T cells and the upregulation of cytotoxic T cell and interferon pathway genes in the coronary artery (50). Apart from that, electron microscopy revealed that cytoplasmic inclusion bodies in ciliated bronchial epithelium aggregate with RNA and viral protein in autopsy specimens (25). This suggests that KD may be triggered by a novel process of acute viral infection *via* the respiratory tract, and then result in dysregulation of immune response (51). And the theory of bacterial superantigens is on the basis of the consistency in clinical presentations among groups, like strawberry tongue and desquamation of hands and feet (52). Some studies have shown that Staphylococcal Toxic Shock Syndrome toxin (TSST-1) and Streptococcal pyogenic toxins may act as superantigens to induce immune responses, which ultimately cause the occurrence of KD (51). Alternatively, it has been suggested that binding of a superantigen to the Vβ region of T cell receptors will initiate the release of immunological mediators, such as IL-6, TNF-α and TGF-β, which are overexpressed in KD patients (53).

It is worth mentioning that, in addition to bacteria and viruses, environmental and other triggers may also explain the seasonality of KD incidence rates. Some studies showed that climate factors seemed to play an essential role in the development of KD. Meanwhile, it was also reported that elevated incidence of KD was correlated with high precipitation and low temperature (54).

### 2.3 Immunity

Several studies have shown that an undiscovered stimulus could trigger inflammatory cascades, with activation of both the innate and adaptive immune systems (55, 56). Vaccination may be one of these triggers.

The innate immune system plays an important role in the development of KD, which may be activated by pathogen-associated molecular patterns (PAMPs) or damage-associated molecular patterns (DAMPs) (56). The most intensely studied cytokines in KD include IL-1, IL-6, IL-8, IL-18, TNF-α and IFN-γ. Among them, IL-1 plays an important role in the pathogenesis of KD. It also has direct inflammatory effects on coronary artery endothelial cells (57). Meanwhile, higher expression of toll-like receptor 2 (TLR2) on the peripheral blood monocytes also assumes that innate immunity is a vital part of the pathogenesis of KD (58).In addition to innate immunity, adaptive immune response has a significant effect on activating inflammatory mechanisms in KD. Striking immunological disorders have been reported in acute KD, including sundry changes in immune cells, marked cytokine cascade reaction, and endothelial cell activation, a slight upward trend of circulating proinflammatory and regulatory T cells (59). A study has noted a growth of IgA producing plasma cells in tissues and coronary artery vascular walls in affected KD patients (60). While the application of IVIG contributed to the resolution of inflammatory and clinical improvement during the acute phase of KD (37). Additionally, tropospheric wind patterns mentioned above may support the claim that the infection pathogens are more likely to penetrate into the body through the respiratory tract and lead to KD.

Recent studies have shown that some perinatal factors could increase the risk of developing KD. They found that a tight association exists between the risk of acquiring KD and maternal age above 35 years, early infant hospitalization and group B streptococcus (GBS) colonization, after a vast array of potential confounders were controlled, like gender, race and birth year. Also, some contend another mechanism exists that early exposure to infections damages the developing immune system and renders it more susceptible to KD. Furthermore, children with immune deficiency have a high risk of KD in their early or later life. However, more studies should be performed to demonstrate this standpoint more definitively.

## 3 The inherent mechanism of miRNAs in Kawasaki disease

### 3.1 MiRNA polymorphism

Genetics is a crucial factor in the occurrence of Kawasaki disease. It was reported that compared to normal population, the incidence could be as high as 10-30 folds in the siblings of Kawasaki patients (61, 62). Various studies have shown that miRNA polymorphism has a tight association with autoimmune disease (63). Di et al. carried out a case-control study comprising 532 children with KD and 623 healthy controls to explore the role of miR-137 polymorphism in KD (17). Although no significant correlation was revealed, the subgroup analysis showed that the rs1625579 T>G polymorphism of miRNA-137 increased the risk of KD in southern Chinese children aged<12 months. Later, several one-center studies demonstrated that the miR-218 rs11134627 A>G, miR-146a rs2910164 C>G, miR-196a2 rs11614913 T >C, miRNA-149 rs2292832 T >C polymorphisms are not associated with the susceptibility of Kawasaki disease (64–67). However, these results still need to be confirmed in a multi-center study involving of larger sample. Besides, emerging reports elucidated the latent relation between miRNA polymorphisms and Kawasaki disease concurrent CALs and miR-196a2 rs11614913 was found to increase the risk of CAD. Zha et al. investigated the apparent relation between CAL risk and miR-146a gene polymorphisms firstly through recruiting 120 patients and 126 healthy populations (68). Wang et al. reported that miRNA -608 rs4919510 G>C polymorphism not only contributed to a higher incidence of KD but also related to CAL risk (69). To overcome the defect of sample capacity, Fu et al. included 318 KD patients with CAAs and 784 KD patients without CAAs (70). The result demonstrated that while miR-499a rs3746444 A>G polymorphism decreased the risk of CAA in KD patients, miR-149 rs2292832 T>C polymorphism increased the risk of CAA in KD patients. These investigations revealed that miRNA polymorphisms affected both the susceptibility of KD and the hazard ratio of CALs.

### 3.2 Regulation of immunity

Kawasaki disease is an auto-immune vasculitis and its central feature includes the activation of both innate and adaptive immune in the patients (71). Immune cells including monocytes, neutrophils and activated T cells were upregulated predominantly in the serum of KD patients. The biogenesis and function of miRNAs play a vital role in the immune response towards Kawasaki disease. Regulatory T cells (Treg) play an important part in ameliorating the inflammatory effect of pathogenic effector T-cells, which result in the damage of coronary artery in KD patients (72). However, several studies reported the dysfunction of Tregs during the acute phase of KD (73). Ni et al. reported that the dysfunction of Treg may be related to the aberrant miR-155/Suppressor of cytokine signaling-1(SOCS-1)/Signal transducer and activator of transcription-5(STAT-5) pathway, interleukin-6(IL-6)/signal transducer and activator of transcription-3(STAT-3)/miR-21 pathway and overexpression of miR-31, which all lead to the modulation of forkhead box protein 3 (FoxP3), the “master regulator” of Treg (74). Neutrophils were also involved in the pathogenesis of KD as well. Li et al. found that the expression of miR-182-5p increased in the serum of patients with CALs compared to those without CALs. Through pathway enrichment and further experiments on human umbilical vein endothelial cells (HUVECs) and neutrophils, they concluded that miR-182-5p could enhance neutrophil infiltration to induce the CAL formation *via* activating the leukocyte trans-endothelial migration pathway (75). Besides, a recent pathway enrichment analysis revealed that the target genes of miR-223-3p are mainly distributed on the T cell receptor and B cell receptor pathways, which are highly relative to immune function (76).

### 3.3 Modulation of inflammatory response

Excrescent inflammation was unavoidable during the pathogenesis of Kawasaki disease. Immune cells participate in the inflammatory process and play an indispensable role. Shimizu et al. proposed that miR-145 could mitigate the inflammation of the arterial walls of KD patients through downregulating the TGF-β pathway, which is essential in triggering the migration of myofibroblasts to the arterial walls, thus aggregating the inflammatory cells (13). Nakaoka et al. proclaimed that has-miR-145-5p and has-miR-320a could affect the function of the monocyte THP-1 through upregulating the expression of TNF-α (77). B10 cells is a set of B cell that could express IL-10 cytokines after differentiation and have strong negative effects on the inflammatory response. Luo et al. suggested that over-expressed miR-27a in B cells of KD patients could stimulate the releasing of TNF-α mediated by monocytes *via* suppressing the B10 cells’ function (78). Through bioinformatics analysis of the serum exosomal miRNAs of KD patients, Zhang et al. predicted that the differentially expressed miR-328, miR-575, miR-134 and mir-671-5p could regulate the class of inflammatory target genes expressed in peripheral blood mononuclear cells (PBMCs) (79). Furthermore, pro-inflammatory cytokines are key factors that influence the process of KD pathogenesis. In detail, IL-6 is crucial in regulating the inflammatory reaction in KD. It was demonstrated that miR-223-3p was overexpressed in acute KD, and in TNF-α treated human coronary artery endothelial cells (HCAECs), miR-223-3p could inhibit the expression of IL-6ST and suppress the expression of pSTAT3 and NF-κB p65 (80). miR-150 -3p was significantly elevated in KD patients, relative studies reported that it could reduce the expression of many pro-inflammatory cytokines *via* the NF-κB pathway (81).

### 3.4 Vascular endothelial dysfunction

One in five KD patients may have CALs, further resulting in severe cardiovascular complications. Vascular endothelial dysfunction, which involves abnormal apoptosis, proliferation and migration, is the main culprit for this vicious process. In KD patients, miR-223 is the most upregulated serum miRNA which could enter vascular endothelial cells (ECs) and thus enter the vascular walls. Chu et al. demonstrated that the overexpressed miR-223 mediated the apoptosis of vascular endothelial cells through like growth factor type 1 receptor (IGF1R)/B-cell lymphoma-2(Bcl2) pathway (16). Compared to healthy controls, miR-125a-5p increases remarkably in KD patients. Li et al. proposed that miR-125a-5p could involve in the HUVECs apoptosis *via* Bcl2 associated X protein (Bax)/Bcl2/caspase-3 pathway targeting MKK7 (82). Similarly, miR-186, which is also elevated in the KD patients’ serum, has been illuminated to induce the apoptosis of ECs *via* activating the mitogen-activated protein kinase (MAPK) pathway through inhibiting SMAD family member 6 (SMAD6) (83). Protein phosphatase 2A(PPA2) plays a key role in maintaining the stability of vascular. Recently, the expression level of miR-133a was detected and the results presented an obvious escalating trend. Luo et al. proclaimed that overexpressed miR-133a in KD patients inhibited the expression of PPP2CA, a catalytic submit of PP2A, thus leading to the cleave of VE-cadherin and vascular dysfunction (84). Endothelial-mesenchymal transition (EndoMT) takes a leading position in coronary arterial wall destruction. According to He et al.’ research, KD sera could impair the Krüppel-like factor 4 (KLF4)/miR-483 axis in ECs and thus induce the upregulation of connective tissue growth factor (CTGF) and activate the EndoMT process (18). However, not all miRNAs contribute to the injury of vessel walls. Several studies have revealed that part of miRNAs could prevent pathogenesis as well. The most famous miR-223-3p has shown a positive regulatory effect on vascular endothelial injury in KD patients (80, 85). Results showed that reduced miR-223-3p in KD patients could promote VSMC differentiation through suppressing platelet-derived growth factor receptor β(PDGFRβ), implying severe coronary injury risk. Through comparing the expression level of miR-223-3p, Zhang et al. revealed that detection of miR-223-3p may conduce to identify patients with CALs risk (86). Rong et al. reported that the upregulated miR-27b in KD patients’ serum could serve as a protective molecular by inhibiting cell proliferation and migration. The underlying mechanism mainly comprises the suppression of the EndoMT process in ECs induced by miR-27b *via* the TGF-β pathway, finally targeting SMAD7 (87). A recent research pointed out that the upregulated miR-197-3p may be involved in inhibiting apoptosis and restraining the proliferation and migration of HCAECs (15). Their work also pointed out that IGF1R and Bcl2, which have been suppressed significantly, are the direct target of miR-197-3p. Besides, Liu et al. explored another underlying mechanism of miR-197-3p in HCAECs. The study demonstrated that miR-197-3p was upregulated *via* TIMP Metallopeptidase Inhibitor 3(TIMP3)/Matrix Metallopeptidase 9(MMP9) signaling pathway, which effectively impedes the ECs migration and invasion (88).

**Figure 2:**
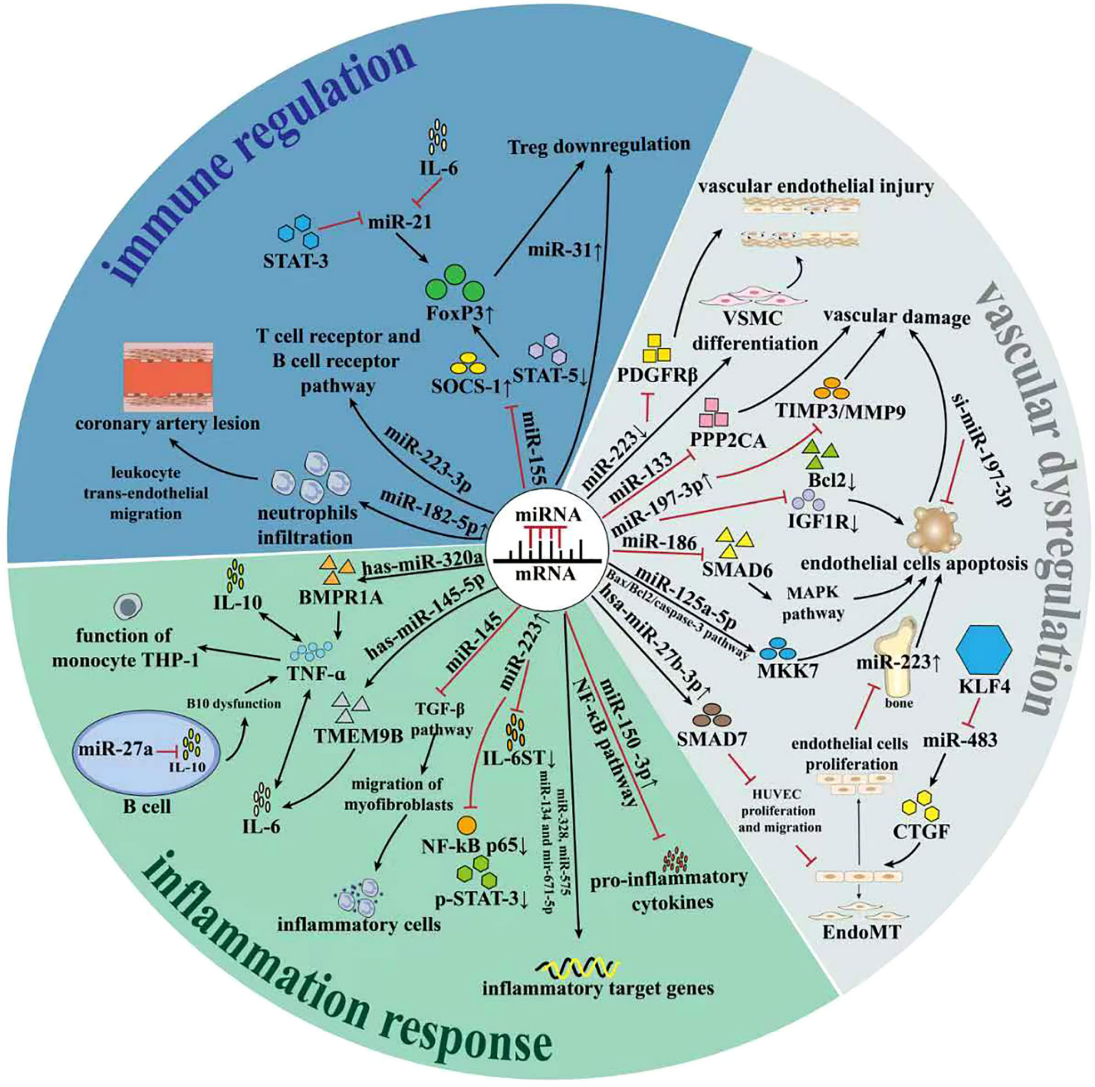
The role of miRNAs in regulation of Kawasaki disease. Recent studies have revealed that miRNAs could play important part in regulation of Kawasaki disease. On one hand, miRNAs involve in the regulation of adaptive immunity, which predominantly affected the progression and prognosis of KD. On the other hand, miRNAs remarkably affected the inflammation response of KD. Besides, miRNAs affected the vascular endothelial dysfunction process *via* influencing related signaling pathway. All these SOCS-1, Suppressor of cytokine signaling; STAT5, signal transducer and activator of transcription 5; KLF4, Krüppel-like factor 4; VSMC, vascular smooth muscle cell; PPP2CA, Protein Phosphatase 2 Catalytic Subunit Alpha; Bcl2, B-cell lymphoma-2;Bax, Bcl2 associated X protein; IGF1R, growth factor type-1 receptor;MKK7, MAP kinase kinase 7; MAPK, mitogen-activated protein kinase; HUVEC, Human Umbilical Vein Endothelial Cell; CTGF, Connective tissue growth factor; Smad7; Endomt, Endothelial-to-mesenchymal transition; PDGFRβ, platelet-derived growth factor receptor β; TMEM9B, Transmembrane protein 9B; BMPR1A, Bone morphogenetic protein receptor 1A; Foxp3, Forkhead box protein 3; TGF-β, transforming growth factor-β;TNF-α, Tumor necrosis factor-α;IL-10, interleukin-17;IL-6, interleukin-6;IL-6ST, interleukin 6 cytokine family signal transducer;THP-1, Tohoku Hospital Pediatrics-1;NF-κB, nuclear factor kappa-B; STAT3, signal transducer and activator of transcription-3.

Surprisingly, miRNAs could dramatically participate in the pathogenesis of Kawasaki disease and represent unique role from several perspectives. We have concluded the underlying mechanism in **Table 1** and draw a graphical abstract in **Figure 2**.

**Table 1:** The role of miRNAs in regulation of KD mechanism.

miRNA	Derivation	Comparative group	miRNA expression	Model	Function	Reference
miR-155	serum	33 KD patients (24 males and 9 females) 14 healthy controls (8 males and 6 females)	↓	FoxP3+ Treg cells in KD patients	Downregulation of Treg cells and induce immune dysfunction	(74)
miR-21			↓			
miR-31			↑			
miR-182-5p	serum	11 KD patients	↑	HL-60 cells	enhance *in vitro* leukocyte infiltration	(75)
miR-223	platelet	242 KD patients	↓	VSMCs	Induce vascular injury *via* VSMCs resolution and differentiation	(86)
	serum	30 KD patients 12 healthy controls	↑	HCAECs	Alleviate vascular endothelial injury	(80)
	Bone-marrow	Male C57BL/56 mice miR-223 Knockout mice chimeric mice	↑	Vascular ECs THP-1 monocyte	Induce ECs injuries	(16)
miR-145	Whole blood	12 acute and convalescent KD patients	↑	KEGG enrichment prediction	Modulate TGF-β signaling pathway in the arterial wall	(13)
miR-200c	serum	12 KD patients 6 healthy controls	↑	KEGG enrichment prediction	Modulation of inflammatory response	(89)
miR-371-5p			↑			
miR-93	PBMCs	23 KD patients 12 healthy controls	↓	Isolated PBMCs	Regulation of VEGF-A expression	(81)
miR150			↓		Regulate NF-κB related inflammation	
miR-145-5p	serum	5 KD patients with CALs 45 KD patients without CALs	↑	THP-1 monocyte	Modulation of inflammatory cytokines expression	(77)
miR-320a			↑			
miR-483	serum	KD patients and controls	↓	HCAECs	Induction of EndoMT and increased CTGF expression	(18)
miR-125a-5p	plasma	30 KD patients 32 healthy controls	↑		Targeting MMK7 to induce ECs apoptosis	(82)
miR-27b	serum	34 acute KD children 42 healthy children 15 febrile children 18 convalescent KD children.	↑	HUVECs	Targeting SMAD7 and TGF-β to suppress the ECs proliferation and migration	(87)
miR-186	serum	25 healthy controls 17 febrile children 11 convalescent children 21 acute KD children	↑	HUVECs	Targeting SMAD6 to induce ECs apoptosis	(83)
miR-197-3p	serum	18 healthy controls 20 convalescent children 32 acute KD children	↑	HCAECs	Regulation of ECs proliferation and migration targeting BCL2 and IGF1R	(15)
	Serum	10 KD patients 10 healthy children	↑	HCAECs	Induce HCAECs damage *via* TIMP3	(88)
miR-27a	PBMCs	23 acute KD children 32 healthy controls	↑	Purified CD19^+^ B cells CD14 monocytes	Inhibition of B10 function	(78)
miR-133a	Plasma	30 acute KD children 30 convalescent children 30 healthy children	↑	HUVECs	Inhibition of PP2CA and induce vascular damage	(84)

KD, Kawasaki disease; FoxP3+, Forkhead box protein P3;Treg, Regulatory T cells; VSMC, vascular smooth muscle cell; HCAEC, human coronary artery endothelial cell; EC, endothelial cell;THP1, Tohoku Hospital Pediatrics-1;KEGG,Kyoto Encyclopedia of Genes and Genomes; VEGF-A vascular endothelial growth factor A; CAL, cardiovascular artery leision; EndoMT, endothelial-mesenchymal transition; CTGF, connective tissue growth factor;MMK7, MAP kinase kinase 7;HUVEC, Human Umbilical Vein Endothelial Cell;SMAD7, SMAD Family Member 7; TGF-β, transforming growth factor-β;SMAD6, SMAD Family Member 6; Bcl2, B-cell lymphoma-2;IGF1R, growth factor type-1 receptor;TIMP3, TIMP Metallopeptidase Inhibitor 3;PBMC, Peripheral blood mononuclear cell;PP2CA, Protein Phosphatase 2 Catalytic Subunit Alpha. ↑, upregulate; ↓, downregulate.

## 4 MiRNAs as diagnostic biomarkers of Kawasaki disease

Currently, the specific diagnostic biomarker of Kawasaki disease is still unclear. Owning to the unavoidable overlaps of the typical symptoms of Kawasaki disease and plenty of other common diseases such as sepsis, bacterial or virus infectious diseases and so on, the diagnosis is confronted with a dilemma. Delayed treatment of Kawasaki disease will cause a high risk of cardiovascular combinations. Therefore, establishing biological biomarkers with high stability, specificity, sensitivity, and noninvasive is of great value for distinguishing Kawasaki disease at an early stage.

Not surprisingly, miRNAs, a small molecule which could be detected easily in urine, blood, and other body fluids using relatively cheap assays have arouse attention (90). What’s more, the existing form of miRNAs are generally stable. Circulating miRNAs could be delivered to high-density lipoprotein or caried by AGO2 protein, and a small proportion of miRNAs could exist in extracellular vesicles (EVs), as well (91, 92). MiRNAs have shown the potential in diagnosing Kawasaki disease in recent years (93). Kuo et al. conducted a high-throughput sequence of miRNAs for Kawasaki disease firstly in 2015. They figured out many differentially expressed miRNAs between KD patients and healthy controls and validated 10 remarkably distinguished miRNAs ultimately (94). Subsequently, Jia et al. reported a class of serum exosomal miRNAs including miR-1246, miR-4436b-5p, and so on as candidate diagnostic biomarkers to distinguish KD from other febrile diseases (95). In that case, through the detection of these miRNAs, doctors could improve the accuracy of differential diagnosis. In addition, miRNAs could identify patients with CAA (96, 97). MiR-let-7i-3p, miR93,7 and many other miRNAs have been reported to be expressed differentially remarkably in CAA patients, which present the importance of isochronous treatments for the complications. On the other hand, miRNAs could predict the therapeutic outcomes of KD to help doctors in identifying the most appropriate therapy for patients. Through conducting clinical trials among KD patients and KD patients after IVIG therapy, Zhang et al. demonstrated that the elevated miR-328 and decreased miR-575, miR-134 and miR-671-5p could predict the possible outcome of IVIG *via* influencing the inflammatory genes (79). We have concluded existing miRNAs that have a certain clinical value in **Table 2**.

**Table 2:** MiRNAs as potential biomarkers for Kawasaki disease.

miRNA	Comparative group	miRNA expression	Reference
miR-1246 miR-4436b-5p miR-197-5p miR-671-5p	20 healthy individuals 20 Kawasaki disease patients 20 Kawasaki disease patients after IVIG treatment 5 adenovirus-infected patients without Kawasaki disease	\	(95)
miR-133a	30 acute KD patients 30 convalescent KD patients	↑	(84)
hsa-let-7b-5p hsa-mir-223-3p hsa-mir-4485 hsa-mir-4644 hsa-mir-4800-5p hsa-mir-6510-50 hsa-mir-765	6 children with Kawasaki disease 6 healthy children	↑	(98)
hsa-mir-33b-3p hsa-mir-4443 hsa-mir-4515		↓	
miR-210-3p miR-184 miR-19a-3p	84 KD patients 29 non-KD febrile patients	↑	(99)
miR-223-3p	16 KD patients 16 pneumonia patients	↑	(76)
miR-let-7i-3p miR-17-3p miR-210-5p	39 healthy children 42 KD patients with CAA 38 CAD patients	↑	(96)
miR-6743-5p miR-1246 miR-6843-5p	45 virus infected patients	↓	
		↑	(100)
miR-1	33 KD patients 15 healthy individuals		
miR-937	25 KD patients with CAD 25 KD patients without CAD	↓	(97)
miR-455-5p	KD children Normal control	↓	(101)
miR-122	150 children with KD 150 children with respiratory infection	↑	(102)
miR-21	100 KD children Febrile children in the same period	↑	(103)
miR-24-3p	74 KD patients 41 healthy controls 36 patients with virus infection	↑	(104)
miR-328	5 healthy children 5 KD patients 5 KD patients following IVIG therapy	↑	(79)
miR-575		↓	
miR-134		↓	
miR-671-5p		↓	

KD, Kawasaki disease; IVIG, intravenous immunoglobulin; CAA, coronary artery aneurism; CAD, cardiovascular diseases. ↑, upregulate; ↓, downregulate.

## 5 The role of miRNA in the clinical application of Kawasaki disease

In recent years, increasing clinical studies revealed the participation of miRNAs in the treatment of Kawasaki disease. Although the established standard therapy could cure most patients, it’s unavoidable to confront insensitive patients and related complications. Fortunately, the differentially expressed miRNAs are conducive to distinguishing the divergence of patients and formulating personalized therapy precepts. In this section, we will summarize the role of miRNAs in the therapy of KD.

### 5.1 First line therapy

#### 5.1.1 IVIG

IVIG has been ranked as the standard therapy for Kawasaki disease since it was come up by Furusho et al. in 1984 (105, 106). It has shown a remarkable effect on reducing the risk of later development of CAL and related cardiovascular complications (42, 107). Although the mechanism of how IVIG plays its role still needs further research, scientific studies have revealed that its efficacy mainly depends on the regulation of immunity (11, 108). Through binding to the Fcγ receptors on immune cells (109) including B cells, T cells, nature killer (NK) cells, neutrophils and mast cells (110, 111), IVIG could reduce the release of cytokines and neutralize pathogenic autoantibodies, infectious antigens and superantigens (57, 112).

Most cases reported excellent outcomes with this traditional therapy. However, approximately 15-20% of patients are insensitive to the IVIG treatment. Besides, plenty of studies demonstrated that IVIG resistant patients are high in CAL and mortality risk (113–115), which indicates that it is crucial for choosing appropriate therapy as soon as possible. In recent years, miRNAs are used to discriminate non-respondent patients from sensitive patients. Zhang et al. conducted a serum miRNAs screen to identify differentially expressed miRNAs between KD patients and those who received following IVIG therapy and the results demonstrated that in the serum of KD patients, the expression level of miR-328 was elevated, and the level of miR-575, miR-134 and miR-671-5p were decreased. These trends were reversed after IVIG treatment, which indicated that miR-575, miR-134 and miR-671-5p could predict the therapeutic effect of IVIG treatment (79). Through the detection of the relative expression levels of miR−21, miR−145, miR−155 and miR−199b−5p in the serum of IVIG-sensitive and IVIG-insensitive patients, Wang et al. reported that the level of miRNA-199b-5p decreased while miR-145 and miR-155 concentrations increased in the IVIG-insensitive group compared to the IVIG-sensitive group. These results suggest that miR-145, miR-155 and miR-199b-5p could be a potential indicator for IVIG (116). A study involves of 80 healthy controls and 102 KD patients focusing on the level of miR-200c and miR-371-5p revealed that miR-200c was found to play a crucial role in the endothelial cell apoptosis process and the anti-inflammation response of the vascular muscle cells (117), indicating the potential of miR-200c in regulating CADs (118). Zhang et al. found that these two miRNAs were much lower in KD patients who are sensitive to IVIG therapy, which could be an extraordinary signal to distinguish the sensitivity of different KD patients (119). Besides, Wang et al. have concluded that the level of miR-937 decreased before treatment with IVIG, while upregulated after IVIG treatment (97). This feature made miR-937 a good biomarker to be applicated in the therapeutic decision. However, it also suggested that some miRNAs such as miRNA-210-3p, miR-184, and miR-19a-3p are good indicators for diagnosing Kawasaki disease, but could not distinguish the patients who are sensitive to IVIG treatment (99).

#### 5.1.2 Aspirin

Acetyl Salicylate Acid (ASA, Aspirin), a classical nonsteroidal anti-inflammatory agent, is widely applied together with IVIG and could reduce the hazard of CAAs 5 folds if used within the first 10 days (41). High-dose of Aspirin is widely used as anti-inflammation medicine, while low-dose of Aspirin is known for its anti-platelet ability. It was found that microRNAs, which perform apparent expression level distinction, could play a role in therapeutic decisions for KD patients. On one hand, miRNAs could take part in the anti-inflammation process of aspirin. MiRNAs play a role in regulating the vessel wall cells. Endothelial injury takes a leading position in KD patients-related CAAs. Recently, a study clarified that through inhibition of NF-κB-dependent miR-155 expression, aspirin could prevent TNF-αtriggered endothelial cell dysfunction (120). Besides, the abnormal proliferation of VSMCs is a common coronary artery pathology that influences the occurrence of plenty of cardiovascular diseases (CVDs). As a vital modulator of the VSMCs, miR-145 is more abundant in the arteries. Guo et al. reported that Aspirin could play anti-proliferation and anti-inflammation effects on VSMCs through regulating miR-145 in the degradation of CD40, a classical tumor necrosis factor (121), indicating that targeting miR-145 could bring better effect for patients undergoing Aspirin treatment. On the other hand, the effect of miRNAs in platelet activation, aggregation and reactivity are also critical in aspirin therapy. Zufferey et al. demonstrated a close relationship between miRNA-135a-5p, miR-204-5p and platelet reactivity in aspirin-treated patients (122). Investigations revealed that KD patients with CALs will lead to a series of platelet activation, which results in a follow-up cascade of vascular damage events (41). MiR-126a can be used to predict vascular damage prognosis. Research in 2013 demonstrated that aspirin could decrease the release of miR-126a induced by platelet activation (123). Cavarretta et al. later compared the ratio of miR-126a between aspirin treatment patients and non-responders, and the results revealed that the level of miR-126a decreased after aspirin treatment (124). Furthermore, cessation of miRNAs, including miRNA-223, miRNA-21, miRNA-150, and miRNA-126, are associated with the regulation of the P2Y12 receptor and thus affect platelet aggregation (125–127). Zhang et al. reported the varied expression of the P2Y12 receptor is correlated with the molecular bias of miR-223, which has abundant expression in platelet and could mediate other immune cells to regulate platelet function in turn *via* platelet-leukocyte interaction (128, 129). Later, Chychel et al. conducted a pilot study and concluded that CAD patients, who are sensitive to dual antiplatelet therapy including aspirin and clopidogrel, have lower miR-223 expression (130). The study by Liu et al. recruited 444 patients and found that the GAS5 polymorphism could be a competitive endogenous RNA for miR-223-3p in the regulation of the P2Y12 expression and highly affect the response of dual anti-platelet therapy (20). However, aspirin resistance, which is tightly associated with an increased risk of cardiovascular-related events, is an unavoidable problem during the whole therapy. To elucidate the mechanism of aspirin resistance, Rosa et al. recruited 60 patients and assessed the role of platelet miRNAs in modulating multidrug resistance protein-4(MRP4), the results confirmed that miR-26b could specifically downregulate the MRP4 expression, while aspirin treatment decrease the miR-26b expression in the circulation of patients (131). Besides, the aspirin-sensitive group exhibited an increased expression level of thromboxane A synthase 1(TBXAS1), while evidence showed that miR-34b-3p could suppress TBXAS1 expression to regulate the aspirin response (132). Therefore, exploring appropriate biomarkers to accomplish personalized treatment is necessary for aspirin therapy. miR-92a has been found to participate in platelet reactivity. Binderup et al. established a miR-92a/PDW score system and applied it in a 50 volunteers’ pilot cohort. The system presented excellent positive and negative values in the prediction of aspirin-resistance patients (21). Two years later, their team developed a validation cohort involving 209 patients and the results concluded that miR-92a and PDW are both upregulated in aspirin-resistance patients (133). Recently, several studies concluded that aspirin-induced platelet aggregation could downregulate the expression of miR-19b-1-5p in patients with higher risk of cardio-cerebrovascular events, indicating that miR-19b-1-5p may serve as an aspirin-sensitivity biomarker (134, 135).

### 5.2 Other therapies

In recent years, other medicines were gradually taken into consideration for Kawasaki disease treatment. Increased level of IL-1 could be a trigger of cytokine storm which leads to awful situation in inflammation. Anakinra, an inhibitor of both IL-1α and IL-1β, has outstanding effect of reducing the fever stage of patients and CALs (136, 137). Besides, Infliximab, a famous TNF-α antagonist, has presented remarkable ability in KD, especially beneficial for drug resistant patients (138).

As an effective vasculitis drug, corticosteroids are widely used for their strong anti-inflammatory and immune regulatory capacities (139). However, it is not the first choice for the treatment of KD and is usually used as adjuvant therapy to improve the therapeutic benefit of IVIG (140). Several studies presented that corticosteroids have a preponderance in refractory patients for reducing the non-response phenomenon and the occurrence of CAA (141, 142). Furthermore, evidence showed that corticosteroids could regulate CADs through modulating the miRNAs expression in vascular. Hao et al. reported the negative modulation role of miR-34b/c in aldosterone-induced VSMC calcification for the first time (143). Besides, Zhang et al. proclaimed that miR-25 is a potential therapy target for corticosteroid-induced VSMCs apoptosis in atherosclerosis disease (144). However, the application of corticosteroids is still under controversy, more studies are needed to determine the role of corticosteroids in KD (145).

Taken together, miRNAs play an indispensable role in formulating appropriate therapy schemes for Kawasaki disease patients. Numerous miRNAs participate in the pathophysiology during the treatment course of Kawasaki disease. Through analyzing the differential expression of miRNAs, it’s easier to identify patients who are drug-resistance and constitute personalized therapy with the least complications.

## 6 Conclusions and perspectives

For decades, researchers are exploring the pathogenesis of KD and its complications on coronary. Although the underlying mechanism remains unknown, the presuppositions are mainly from 3 aspects: genetics, infectious and immunity. Recently, miRNA was noticed for its modulation ability of genes at the post-transcriptional level through specific miRNA-mRNA networks. Lots of miRNAs have shown a unique and extraordinary role in the pathogenesis of KD, including the injury of vascular endothelial cells and unusual immune cells. MiRNAs have been reported to contribute to the apoptosis of endothelial cells and the proliferation of VSMCs, while affecting both innate immunity and adaptive immunity by regulating the secretion and abnormal release of immunity cells, including Treg cells, B cells and peripheral monocytes. Without doubt, miRNAs are critical molecules for the occurrence and development of Kawasaki disease. Furthermore, the occurrence of CAAs is a dangerous but inevitable phenomenon in Kawasaki patients, especially under delayed treatment. It contributes to the incidence of related cardiovascular events and mortality, which induce health hazards uncontrollably. Previous studies have mentioned that the coronary artery wall of Kawasaki disease patients was infiltrated with a variety of immune cells including macrophages, neutrophils, CD8^+^ T cells and so on. These cells could secret pro-inflammatory cytokines such as TNF-α, IL-1β, which dramatically promote the development of CAAs. Interestingly, our review firstly shed light on the role of miRNAs on the pathogenesis and prognosis of subsequent CAAs of KD patients. The miRNAs polymorphism induces the variation of risk of CAAs in population. In addition, through differential expression, miRNAs could modulate immune activity of KD patients. For example, miR-182-5p could mediate the neutrophil infiltration process. Most importantly, the influence of miRNAs on vascular endothelial cells could not be overlooked. Studies revealed that plenty miRNAs could lead to vessel walls injury while a small proportion of miRNAs could play protective effect such as miR-223p, miR-27b. Although controversial conclusions are reported in contemporary studies, one thing is for sure, miRNAs could not only influence on the KD development, the value of miRNAs on CAAs are profound as well.

Except for influencing the pathogenesis of KD, miRNA is an extraordinary biomarker for the diagnosis and classification of KD patients. Through RNA sequence analysis, plenty of miRNAs showed predominant expression levels in KD patients compared to healthy individuals. Besides, differential expression of miRNA was observed in patients’ groups with or without risk of development of CALs, as well. It is worth noting that there is still a huge gulf from “bench to bedside”. The extraction efficiency of miRNA is relatively low, which induce higher requirement of specificity and sensitivity for miRNAs and create a dilemma in application. However, more efficient extraction and detection techniques have been invented such as droplet PCR, Ion-Exchange Nanodetector and so on (146). These advanced methods could not only elevate the sensitivity but also reduce the processing time. Besides, clinical trials nowadays are basically confronted with the drawback of limited samples. Further and bigger multi-center experiments are in need to boost the maturation of the application of miRNAs in clinical patients, which could improve the specificity in some extent.

Nowadays, the combination of IVIG and Aspirin has been listed as the leading treatment for Kawasaki disease. However, though the standard therapy is proved effective in the past, numerous problems regarding resistance and prognosis have aroused. With more comprehensive understanding of miRNA, plenty of studies revealed that miRNAs are involved in the pathophysiology during the treatment and development of drug resistance, indicating their potential as therapeutic targets for KD. Furthermore, the level of differentially expressed miRNAs could be propitious for discriminating the patients and formulating appropriate therapy.

In conclusion, miRNAs could be involved in the pathogenesis, treatments and prognosis prediction of Kawasaki disease.

## Author contributions

Conceptualization, JZ and PY and XL and DZ; writing—original draft preparation, JX and YX; writing—review and editing, PY and JZ and JL; project administration, PY and JL; funding acquisition, JL and PY. All authors have read and agreed to the published version of the manuscript.

## Funding

This work was supported by the Natural Science Foundation in Jiangxi Province grant [grant numbers No.202002BAB216022 to JZ, No.20192ACBL21037 and No.202004BCJL23049 to PY]; the National Natural Science Foundation of China [grant number No. 82160371 to JZ, and No. 82100869 to PY]; Youth science foundation of Jiangxi (20192ACBL21036).

## Acknowledgments

We sincerely appreciated the guidance from our tutors and the work of every member in our team. The graphical abstracts were created with BioRender software (https://BioRender.com).

## Conflict of interest

The authors declare that the research was conducted in the absence of any commercial or financial relationships that could be construed as a potential conflict of interest.

## Publisher’s note

All claims expressed in this article are solely those of the authors and do not necessarily represent those of their affiliated organizations, or those of the publisher, the editors and the reviewers. Any product that may be evaluated in this article, or claim that may be made by its manufacturer, is not guaranteed or endorsed by the publisher.

## Glosssary

**Table:**

KD	Kawasaki disease
miRNAs	microRNAs
TGF-β	transforming growth factor-β
NF-κB	nuclear factor kappa-B
CAA	coronary artery aneurism
BLK	B cell lymphoid kinase
TGFB2	Transforming Growth Factor Beta 2
IVIG	intravenous immunoglobulin
HLA	human leukocyte antigen
ITPKC	inositol 1
4	5-Trisphosphate kinase-C
IL-2	interleukin-2
CASP3	caspase3
AICD	activation induced cell death
NFAT	nuclear factor of activated T cells
GWAS	Genome wide association study
IL-17	interleukin-17
FCGR2A	Fc fragment of IgG receptor IIa
TSST-1	Toxic Shock Syndrome toxin
ICs	immune complexes
STING	stimulator of interferon genes
IL-6	interleukin-6
TNF-α	transforming growth factor-α
IFN-γ	interferon-γ
PAMPs	pathogen-associated molecular patterns
DAMPs	damage-associated molecular patterns
TLR2	toll-like receptor2
GBS	group B streptococcus
CAL	coronary artery lesion
Treg	Regulatory T cell
SOCS-1	Suppressor of cytokine signaling-1
STAT-5	signal transducer and activator of transcription-5
STAT-3	signal transducer and activator of transcription-3
FoxP3	Forkhead box protein P3
HUVEC	Human Umbilical Vein Endothelial Cell
IL-6ST	interleukin 6 cytokine family signal transducer
ECs	endothelial cells
IGF1R	growth factor type-1 receptor
Bcl2	B-cell lymphoma-2
Bax	Bcl2 associated X protein
MKK7	MAP kinase kinase 7
MAPK	mitogen-activated protein kinase
SMAD6	SMAD Family Member 6
SMAD7	SMAD Family Member 7
PPA2	pyrophosphatase 2
PPP2CA	Protein Phosphatase 2 Catalytic Subunit Alpha
PP2A	Protein Phosphatase
EndoMT	endothelial-mesenchymal transition
KLF4	Krüppel-like factor 4
CTGF	connective tissue growth factor
PDGFRβ	Platelet Derived Growth Factor Receptor Beta
VSMC	vascular smooth muscle cell
HCAEC	human coronary artery endothelial cell
TIMP3	TIMP Metallopeptidase Inhibitor 3
MMP9	Matrix Metallopeptidase 9
NK	nature killer
CAD	cardiovascular associated disease
ASA	acetyl salicylate acid
CVDs	cardiovascular diseases
MRP4	multidrug resistance protein-4
TBXAS1	thromboxane A synthase
PDW	Platelet distribution width
